# Phenotypical differences and thrombosis rates in secondary erythrocytosis versus polycythemia vera

**DOI:** 10.1038/s41408-021-00463-x

**Published:** 2021-04-15

**Authors:** Eliane Nguyen, Michaël Harnois, Lambert Busque, Shireen Sirhan, Sarit Assouline, Ines Chamaki, Harold Olney, Luigina Mollica, Natasha Szuber

**Affiliations:** 1 Department of Hematology, Hôpital Maisonneuve-Rosemont, Montreal, Canada; 2 Université de Montréal, Montreal, Canada; 3 Groupe Québécois de Recherche sur la LMC et NMP (GQR LMC-NMP)/Chronic Myeloid Leukemia and Myeloproliferative Neoplasms Quebec Research Group, Montreal, Canada; 4 Segal Cancer Center, Jewish General Hospital, Montreal, Canada; 5 Department of Oncology, McGill University, Montreal, Canada; 6 Department of Hematology, Hôpital du Sacré-Cœur de Montréal, Montreal, Canada; 7 University of Montreal Health Centre, Montreal, Canada

**Keywords:** Myeloproliferative disease, Myeloproliferative disease

Dear Editor,

Erythrocytosis is a common condition and an increasingly frequent reason for consultation in hematology. Since the inception of the 2016 World Health Organization (WHO) 2016 criteria, in lowering the hemoglobin (Hb) and hematocrit (Hct) diagnostic thresholds to 165 g/L and 49% in men, and 160 g/L and 48% in women, respectively^1^, it has been estimated that 4.1% of unselected males (outpatients) have Hb levels exceeding these values^2^. With only a minority of these having polycythemia vera (PV)^3^, hematologists are witnessing a new preponderance of referrals for secondary erythrocytosis (SE) which has yielded novel and significant diagnostic and therapeutic challenges. While the classic coupling of *JAK2*–positive/subnormal serum erythropoietin (Epo) greatly increases the likelihood of PV diagnosis^4,5^, those not strictly fulfilling these criteria represent a heterogeneous population for whom a systematic approach has been difficult to establish^6^. Though efforts have been made to operationally discriminate between the various forms of erythrocytosis, data comparatively assessing SE and PV populations are scarce^7,8^. These support different clinical profiles^7,8^, while reports of outcomes, including thrombosis, have been inconsistent^9,10^. Furthermore, little information exists on how these populations are managed in a real world setting, and it may be speculated that SE cohorts are subject either to under or over investigating and treatment. We conducted a direct comparison of clinical and laboratory features, outcomes, diagnostic workup, and treatment patterns in cohorts with SE vs WHO-defined PV.

## Methods

This multicentric retrospective study included PV patients enrolled in a pan-provincial registry of the chronic myeloid leukemia (CML) and myeloproliferative neoplasms (MPN) Quebec Research Group (GQR LMC-NMP) and has Institutional Review Board approval. PV (multicentric) and SE (Maisonneuve-Rosemont Hospital) patients diagnosed between January 1999 and December 2019 were considered. PV diagnosis was per the WHO 2016 criteria^1^, with the exemption of bone marrow biopsies (not performed in all). Secondary erythrocytosis was defined as sustained elevation in hemoglobin and/or hematocrit above WHO-thresholds for PV with negative *JAK2*V617F mutation testing and non-subnormal serum Epo levels (if subnormal, then bone marrow sampling to exclude *JAK2* exon 12 mutations and/or endogenous erythroid colony testing, EEC, were performed). Serum Epo levels were measured using standard ELISA assay methods^11^ and stratified according to reference values (range 3–30 mIU/mL). Levels obtained >3 months from diagnosis and/or measurements from non therapy-naive patients were excluded. *JAK2* mutational screen and risk-stratification for PV were according to convention^5^. EEC assays, extensively used and validated in our center, were performed on peripheral blood and/or bone marrow according to standard methods^12^. Laboratory and clinical data corresponding to the time of diagnosis were abstracted, including cardiovascular risk factors as well as data on SE etiology, treatment history, complications (thrombosis, fibrotic/leukemic transformation), and survival. Only major arterial and venous thrombotic events were considered, as previously defined^13^. Standard statistical methods were used to assess variables across etiologically-stratified erythrocytosis groups (PV vs SE) with *p* values < 0.05 considered significant. JMP® Pro 14.0.0 software was used for all analyses (SAS Institute, Cary, NC, USA).

## Results and discussion

### Clinical characteristics

Laboratory and clinical features of 102 informative cases of erythrocytosis, stratified by etiology: SE (*n* = 36) vs PV (*n* = 66), are presented in Table 1. Median age was significantly lower in the SE (57 years old; range 19–76) vs PV group (63.5 years old; range 20–89) (*p* = 0.005), with a preponderance of males (75% in SE vs 45% PV; *p* = 0.004). Median serum Epo levels were normal in the SE cohort (10.3 mIU/mL; range <1–148) vs subnormal in PV patients (2.3 mIU/mL; range <1–14.1) (*p* < 0.0001). Epo level categories were also significantly different between the groups: subnormal/normal/high levels were recorded in 3/26/7 of SE and 43/13/0 of PV patients, respectively (p < 0.0001). At diagnosis, SE vs PV patients had significantly lower baseline platelet (191 vs 417 × 10^9^/L; range 125–476 vs120–995) and leukocyte counts (7.2 vs 10 × 10^9^/L, range 4.1–15.1 vs 4.5–20.5) as well as lactate dehydrogenase (LDH) levels (182 vs 247 U/L, range 126–316 vs 157–874) (*p* < 0.0001 for all). Though no differences were observed in median Hb values (*p* = 0.69), a significantly lower proportion of SE patients displayed Hct >55% at diagnosis compared to PV (25% vs 48%, *p* = 0.02). Palpable splenomegaly was also significantly less frequent in SE (*n* = 2, 6%) vs PV patients (*n* = 14, 23%) (*p* = 0.02). Lifestyle and comorbidity analysis revealed higher median body mass index (BMI) (33.4 vs 26) and an excess of obese patients (75% vs 17%) and active smokers (31% vs 12%) in SE vs PV groups respectively (*p* = 0.02–0.03), while no appreciable differences were found in the prevalence of hypertension, diabetes, hyperlipidemia, pulmonary disease, or sleep apnea (*p* = 0.18–0.88).

**Table 1:** Clinical characteristics, outcomes, and treatment patterns of patients with secondary erythrocytosis versus World Health Organization-defined polycythemia vera.

Variables	All patients (*n* = 102)	Secondary erythrocytosis patients (*n* = 36)	PV patients (*n* = 66)	*P* value
Age at diagnosis, years; median (range)	61 (19–89)	57 (19–76)	63.5 (20–89)	**0.005**
Males; *n* (%)	57 (56)	27 (75)	30 (45)	**0.004**
Serum Epo levels, mIU/mL; median (range) “N” evaluable = 92 (90%)	2.9 (<1–148)	10.3 (<1–148)	2.3 (<1–14.1)	**<0.0001**
Serum Epo level categories: Subnormal/Normal/High; *n* (%)	46/39/7 (45/38/7)	3/26/7 (8/72/19)	43/13/0 (77/23/0)	**<0.0001**
Hemoglobin, g/L; median (range) “N” evaluable = 100 (98%)	178.5 (151–223)	176 (160–206)	179 (151–223)	0.89
Hematocrit; median (range) “N” evaluable = 99 (97%)	53.7 (44.6–70.2)	52.9 (48–60)	54.2 (44.6–70.2)	0.12
Hematocrit>55%; *n* (%) “N” evaluable = 99 (97%)	39 (39)	9 (25)	30 (48)	**0.02**
Platelets, × 10^9^/L; median (range) “N” evaluable = 101 (99%)	326 (120–995)	191 (125–476)	417 (120–995)	**<0.0001**
Platelets > 450 × 10^9^/L; *n* (%) “N” evaluable = 101 (99%)	27 (27)	1 (3)	26 (40)	**<0.0001**
Leukocytes, × 10^9^/L; median (range) “N” evaluable = 101 (99%)	8.9 (4.1–20.5)	7.2 (4.1–15.1)	10 (4.5–20.5)	**<0.0001**
Leukocytes > 11 × 10^9^/L; *n* (%) “N” evaluable = 101 (99%)	24 (24)	1 (3)	23 (35)	**<0.0001**
LDH at diagnosis, U/L; median (range) “N” evaluable = 72 (71%)	216 (126–874)	182 (126–316)	247 (157–874)	**<0.0001**
Palpable splenomegaly at diagnosis; *n* (%) “N” evaluable = 97 (95%)	16 (16)	2 (6)	14 (23)	**0.02**
*Driver mutation* status “N” evaluable = 102 (100%)	–	–	–	**<0.0001**
*JAK2V617F*; *n* (%)	61 (60)	0	61 (92)	
*JAK2 exon 12*; *n* (%)	3 (3)	0	3 (5)	
*JAK2V617F* unmutated^a^*; n* (%)	38 (37)	36 (100)	2 (3)	
*JAK2* allele frequency; median (range) “N” evaluable = 49 (74%)	–	–	57.3 (3.67–95.3)	–
Endogenous erythroid colony testing	–	–	–	**0.0003**
Performed*; n* (%)	20 (20)	18 (50)	2 (3)	
Negative result*; n* (%)	18 (90)	18 (100)	0 (0)	
Bone marrow aspirate and biopsy; *n* (%)	37 (36)	11 (31)	26 (39)	0.37
Conventional PV risk stratification “N” evaluable = 66 (100%)	–	–	–	–
Low risk; *n* (%)	–	–	13 (20)	–
High risk; *n* (%)	–	–	53 (80)	–
Body mass index; median (range) “N” evaluable = 68 (67%)	26.5 (17.1–51.6)	33.4 (27–34.8)	26 (17.1–51.6)	**0.02**
Active smoker; *n* (%)	19 (18)	11 (31)	8 (12)	**0.03**
Hypertension; *n* (%)	43 (42)	12 (33)	31 (47)	0.18
Diabetes; *n* (%)	17 (17)	8 (22)	9 (14)	0.27
Obesity; *n* (%) “N” evaluable = 68 (67%)	14 (21)	3 (75)	11 (17)	**0.02**
Hyperlipidemia	33 (32)	10 (28)	23 (35)	0.46
Pulmonary disease	12 (12)	4 (11)	8 (12)	0.88
Obstructive sleep apnea	15 (15)	4 (11)	11 (17)	0.44
Etiologies of secondary erythrocytosis; *n* (%) “N” evaluable = 36 (100%)	–		–	–
Idiopathic		18 (50)		
Smoking/COPD		8 (22.2)		
Smoking + additional factor^b^		3 (8.3)		
Obstructive sleep apnea		2 (5.6)		
Polycystic kidneys		2 (5.6)		
Sleep apnea + polycystic kidneys		1 (2.7)		
Sleep apnea + liver lesion		1 (2.7)		
Post renal transplant		1 (2.7)		
Therapy regimens (exposure, ever); *n* (%)				
Phlebotomy	67 (66)	17 (47)	50 (76)	**0.004**
Aspirin	82 (80)	20 (56)	62 (93)	<**0.0001**
Cytoreduction	58 (57)	0 (0)	58 (88)	<**0.0001**
*hydroxyurea*			58 (88)	
*ruxolitinib*			9 (14)	
*anagrelide*			4 (6)	
*interferon*			0 (0)	
Fibrotic transformations; *n* (%) “N” evaluable = 66 (100%)	6 (6)	–	6 (9)	–
Leukemic transformations; *n* (%) “N” evaluable = 66 (100%)	0	–	0	–
Follow-up in months; median (range)	41 (0.7–238)	10.2 (0.7–47)	68.2 (11–238)	<**0.0001**
Deaths; *n* (%)	5 (5)	0 (0)	5 (8)	**0.03**

*WHO*, World Health Organization, *PV*, polycythemia vera, *Epo*, erythropoietin, *ND*, non detectable, *LDH*, lactate dehydrogenase, *JAK2*, Janus kinase 2, *COPD*, chronic obstructive pulmonary disease.

^a^PV patients in the *JAK2V617F* unmutated category (*n* = 2) either had unmutated exon 12 (*n* = 1) or were not tested (*n* = 1); both had subnormal Epo and positive endogenous erythroid colony testing + / − bone marrow biopsy sampling consistent with the diagnosis of PV. SE patients in the *JAK2V617F* unmutated category included patients who had negative exon 12 testing (*n* = 10) vs exon 12 not performed (*n* = 26). All of the latter had normal/high Epo levels with the exception of one patient whose EEC testing was negative. Bone marrow sampling or EEC testing was performed in a proportion of the remainder (*n* = 9) to exclude PV.

^b^Other combined etiologies included: drug use (invokana), endocrine (nature unspecified), and sleep apnea.

Bold values indicate statistically significant values.

Etiologies of erythrocytosis in the SE cohort were reported as idiopathic in 50% (*n* = 18), smoking/chronic obstructive pulmonary disease (COPD) in 22.2% (*n* = 8), smoking-related plus additional factors in 8.3% (*n* = 3), sleep apnea and polycystic kidney disease in 5.6% (*n* = 2) each, or combination of the latter two in 2.7% (*n* = 1), sleep apnea plus liver lesion in 2.7% (*n* = 1), and post kidney transplant erythrocytosis in 2.7% (*n* = 1) (Table 1). Extensive investigations, including abdominal and lung imaging, sleep studies, and directed testing according to patients’ comorbidities and symptoms, in order to rule out benign or neoplastic causes of polycythemia, are outlined in Supplemental Table 1. A significant portion (*n* = 18, 50%) of SE patients underwent EEC assays, and nearly as many SE (*n* = 11, 31%) as PV patients (*n* = 26, 39%) were exposed to bone marrow sampling (*p* = 0.37) as part of their workup. While PV patients had higher overall treatment rates, SE patients were not infrequently exposed to phlebotomy (47% vs 76% PV, *p* = 0.004) and aspirin therapy (56% vs 93% PV, *p* < 0.0001), though none underwent cytoreduction, which was exclusively directed at PV patients (*n* = 58, 88%) (Table 1). Phlebotomies were performed for multiple reasons in SE, notably to target empirically a hematocrit below 55% (Supplemental Table 1). Rates of fibrotic transformation in the PV cohort were low (*n* = 6, 9%) and no leukemic transformations were recorded. Median follow-up was 10.2 and 68.2 months with (0%) and 5 (8%) deaths recorded in SE vs PV cohorts, respectively (Table 1). We, additionally, performed analyses specifically comparing laboratory characteristics of idiopathic erythrocytosis (IE) vs PV patients (Supplemental Table 2). Notably, *none* of the IE patients had leukocyte counts >11 × 10^9^/L, platelet counts >450 × 10^9^/L, or subnormal EPO levels (*p* = 0.0001-0.0002). While being cognizant of the small sample (*n* = 18), the combination of the aforementioned laboratory criteria, with the addition of LDH in normal range, has a sensitivity of 94% and a specificity of 96% to identify IE (positive predictive value of 85%; negative predictive value of 98%).

### Thrombotic events

Details of thrombotic events occurring in SE vs PV populations are presented in Table 2. Of note, both cohorts had similar rates of arterial or venous thrombosis at/prior to diagnosis including events in 9 (25%) SE and 19 (29%) PV patients (*p* = 0.68), while thrombotic events after diagnosis were less frequent in the SE (*n* = 0, 0%) vs PV cohort (*n* = 9, 14%; *p* = 0.004). At the time of thrombosis (post diagnosis), 33.3% of PV patients were found to have *Hct* >45%. Supplemental Table 3 comparatively evaluates patients with or without a history of thrombosis at any time. Variables clustering significantly with the thrombosis cohort included older age (*p* = 0.001), hypertension (*p* < 0.0001), diabetes (*p* = 0.01), obesity (*p* = 0.01), and hyperlipidemia (*p* < 0.0001).

**Table 2:** Details of thrombotic events occurring in secondary erythrocytosis versus World Health Organization-defined polycythemia vera patients prior to/at diagnosis and after diagnosis.

Thrombotic event	All patients (*n* = 102)	Secondary erythrocytosis patients (*n* = 36)	PV patients (*n* = 66)	*P* value
Any thrombosis at or prior to diagnosis; *n* (%)	28 (27)	9 (25)	19 (29)	0.68
Arterial thrombosis; *n* (%)	23 (23)	7 (19)	16 (24)	0.58
Cerebrovascular	12 (52)	1 (14)	11 (69)	**0.04**
Acute coronary syndrome (MI/angina)	5 (22)	2 (29)	3 (19)	
Peripheral artery disease	5 (22)	3 (43)	2 (13)	
Splanchnic	1 (4)	1 (14)	0 (0)	
Venous thrombosis; *n* (%)	6 (6)	2 (6)	4 (6)	0.92
DVT	4 (67)	2 (100)	2 (50)	0.35
PE	1 (17)		1 (25)	
DVT + PE	1 (17)		1 (25)	
Timing of event prior to diagnosis in years; median (range)	4.7 (0–46.8)	5.1 (0.6–12.4)	4.4 (0–46.8)	0.82
Any thrombosis after diagnosis; *n* (%)	9 (9)	0 (0)	9 (14)	**0.004**
Arterial thrombosis after diagnosis; *n* (%)	7 (7)	0 (0)	7 (11)	**0.01**
Cerebrovascular	3 (43)	0 (0)	3 (43)	
Acute coronary syndrome (MI/angina)	4 (57)	0 (0)	4 (57)	
Venous thrombosis after diagnosis; *n* (%)	3 (3)	0 (0)	3 (5)	0.10
DVT	1 (33.3)		1 (33.3)	–
PE	1 (33.3)		1 (33.3)	
Ocular	1 (33.3)		1 (33.3)	
Hemoglobin at time of thrombosis after diagnosis, g/L; median (range) “N” evaluable = 9 (100%)	140 (117–204)	–	140 (117–204)	–
Hematocrit at time of thrombosis after diagnosis; median (range) “N” evaluable = 9 (100%)	42 (34–61.1)	–	42 (34–61.1)	–
Hematocrit > 45% at time of thrombosis after diagnosis; *n* (%) “N” evaluable = 9 (100%)	3 (33.3)	–	3 (33.3)	–
Platelets at time of thrombosis after diagnosis, x 10^9^/L; median (range) “N” evaluable = 9 (100%)	280 (164–840)	–	280 (164–840)	–
Platelets > 450 × 10^9^/L at time of thrombosis after diagnosis; *n* (%) “N” evaluable = 9 (100%)	2 (22)	–	2 (22)	–
Leukocytes at time of thrombosis after diagnosis, x 10^9^/L; median (range) “N” evaluable = 9 (100%)	6.4 (3.8–33.9)	–	6.4 (3.8–33.9)	–
Leukocytes > 10 × 10^9^/L at time of thrombosis after diagnosis; *n* (%) “N” evaluable = 9 (100%)	2 (22)	–	2 (22)	–

*WHO*, World Health Organization, *PV*, polycythemia vera, *MI*, myocardial infarction, *DVT*, deep vein thrombosis, *PE*, pulmonary embolism.

Bold values indicate statistically significant values.

The diagnosis and the management of erythrocytosis are becoming increasingly complex, particularly in the case of *JAK2* unmutated subjects. How thorough investigations and therapeutic interventions should be is an issue hematologists must now commonly be confronted with. Although limitations of the current study included its retrospective nature, limited sample size, preclusion of red cell mass testing, and more circumscribed follow-up in SE subjects, it disclosed salient phenotypic differences between PV and SE cohorts, and exposed previously unreported management patterns in SE patients. SE patients were significantly younger, more likely to be male, were active smokers, and obese, with normal–high Epo, normal LDH, and exceedingly rare leukocytosis and thrombocytosis, ostensibly discriminating them phenotypically, from their PV counterparts. Consistent with previous reports^14^, approximately one quarter of PV patients in our cohort had normal–high Epo levels, underscoring that while less connotative, these levels do not preclude PV diagnosis; just as rarely, subnormal Epo levels may be detected in SE patients.

Importantly, rates of thromboembolic events, prior to diagnosis, were comparable in both PV and SE populations, suggesting that from the standpoint of thrombosis, SE may not be as benign as some reports have intimated^9,15^. While post-diagnosis thrombotic rates appeared lower in SE patients, limited follow-up in this cohort precludes accurate interpretation. Furthermore, regardless of erythrocytosis etiology, classic cardiovascular risk factors (age, hypertension, obesity, etc.) significantly clustered with thrombosis risk, emphasizing the importance of controlling these risk factors in PV (in addition to Hct targets) as well as SE patients.

Notably, a third of SE patients underwent bone marrow sampling with patent low-yield, raising the question of how to better discern patients that require this not entirely anodyne intervention. Similarly, from a therapeutic perspective, the fact that a significant proportion of SE patients were exposed to phlebotomy and aspirin, both of which remain controversial in secondary etiologies^6^ and may not be completely innocuous, highlights the need for formal trials of these therapies in SE. Finally, while further studies are needed to more comprehensively address the unmet needs relevant to SE, the current observations call for a reappraisal of workup and management practices, and raise concerns for potentially increased thrombotic complications in this understudied population.

## Supplementary information

Supplemental Table 1

Supplemental Table 2

Supplemental Table 3
