# Supplementary material for: Phenotypical differences and thrombosis rates in secondary erythrocytosis versus polycythemia vera
Source: Blood Cancer J. 2021 Apr 15;11(4):75. doi: 10.1038/s41408-021-00463-x (PMC8050282; doi:10.1038/s41408-021-00463-x)
Supplement: Supplementary file 1 — Supplemental Table 1 [file 41408_2021_463_MOESM1_ESM.docx]

**Supplemental Table 1. Investigations and treatments directed at secondary erythrocytosis patients**

| **Investigations** | **Secondary erythrocytosis patients**  *(n=36)* |
| --- | --- |
| Abdominal imaging; *n* (%)   - Any - Abdominal ultrasound - Abdominal scan - Magnetic resonance imaging (MRI) | 29 (81)  16 (44)  14 (39)  5 (14) |
| Lung imaging; *n* (%)   - Any - Chest X-ray - Thoracic scan | 23 (64)  9 (25)  15 (42) |
| Sleep study; *n* (%)  Respirology consultation; *n* (%) | 11 (31)  5 (14) |
| Other investigations; *n* (%)   - Head imaging (scan or MRI) - Erythropoietin receptor mutation | 5 (14)  4 (11) |
| Therapy regimens (exposure, ever); *n* (%)   - Phlebotomy - Aspirin - Cytoreduction | 17 (47)  20 (56)  0 (0) |
| Reasons for phlebotomy; *n* (%)  *“N” evaluable = 16/17 phlebotomized (94%)*   - Empirical target of hematocrit < 45% - Target of hematocrit < 55%   - Symptomatic   - Asymptomatic - Peri-operative setting - Not mentioned | 2 (12.5)  11 (69)  - 2 (12.5)  - 9 (56)  1 (6)  2 (12.5) |
