# Supplementary material for: Phenotypical differences and thrombosis rates in secondary erythrocytosis versus polycythemia vera
Source: Blood Cancer J. 2021 Apr 15;11(4):75. doi: 10.1038/s41408-021-00463-x (PMC8050282; doi:10.1038/s41408-021-00463-x)
Supplement: Supplementary file 2 — Supplemental Table 2 [file 41408_2021_463_MOESM2_ESM.docx]

**Supplemental Table 2.** **Characteristics of patients with idiopathic erythrocytosis versus World Health Organization-defined polycythemia vera**

| **Variables** | **All patients**  **(*n*=84)** | **Idiopathic erythrocytosis**  **(*n*=18)** | **PV**  **(*n*=66)** | **P value** |
| --- | --- | --- | --- | --- |
| Age at diagnosis, years; median (range) | 62 (19-89) | 46 (19-76) | 63.5 (20-89) | **0.003** |
| Males; *n* (%) | 45 (54) | 15 (83) | 30 (45) | **0.003** |
| Serum Epo levels, mIU/mL; median (range)  “N” evaluable=74 (88%) | 2.5 (<1-24) | 10.2 (3.5-24) | 2.3 (<1-14.1) | **<0.0001** |
| Serum Epo level categories:  Subnormal/Normal/High; *n* (%) | 43/28/3  (58/38/4) | 0/15/3  (0/83/17) | 43/13/0  (77/23/0) | **<0.0001** |
| Hemoglobin, g/L; median (range)  “N” evaluable=82 (98%) | 178.5 (151-223) | 176 (160-188) | 179 (151-223) | 0.62 |
| Hematocrit; median (range)  “N” evaluable=81 (96%) | 53.7 (44.6-70.2) | 52.6 (48-57.7) | 54.2 (44.6-70.2) | 0.09 |
| Hematocrit>55%; *n* (%) | 32 (40) | 2 (11) | 30 (48) | **0.003** |
| Platelets, x 10^9^/L; median (range)  “N” evaluable=83 (99%) | 344 (120-995) | 187 (130-344) | 417 (120-995) | **<0.0001** |
| Platelets>450 x10^9^/L; *n* (%) | 26 (31) | 0 (0) | 26 (40) | **<0.0001** |
| Leukocytes, x 10^9^/L; median (range)  “N” evaluable=83 (99%) | 9.2 (4.1-20.5) | 6.7 (4.1-9.7) | 10 (4.5-20.5) | **<0.0001** |
| Leukocytes>11x10^9^/L; *n* (%) | 23 (28) | 0 (0) | 23 (35) | **0.0002** |
| LDH at diagnosis, U/L; median (range)  “N” evaluable=57 (68%) | 235 (126-874) | 174 (126-316) | 247 (157-874) | **<0.0001** |
| Palpable splenomegaly at diagnosis; *n* (%)  “N” evaluable=79 (94%) | 15 (19) | 1 (5) | 14 (23) | 0.07 |
| Leukocytes<11x10^9^/L AND platelets <450 x10^9^/L AND serum Epo normal/high  “N” evaluable=83 (99%) | 24 (29) | 18 (100) | 6 (9) | **<0.0001** |
| Leukocytes<11 AND platelets <450 x10^9^/L AND serum Epo normal/high AND LDH within normal range; U/L  “N” evaluable=64 (76%) | 20 (31) | 17 (94) | 3 (6) | **<0.0001** |
| Endogenous erythroid colony testing  Performed*; n* (%)  Negative result*; n* (%) | 11 (13)  9 (82) | 9 (50)  9 (100) | 2 (3)  0 (0) | **0.001** |
| Bone marrow aspirate and biopsy; *n* (%) | 31 (36) | 5 (28) | 26 (39) | 0.36 |
| Active smoker; *n* (%) | 8 (10) | 0 (0) | 8 (12) | **0.04** |
| Hypertension; *n* (%) | 37 (44) | 6 (33) | 31 (47) | 0.3 |
| Diabetes; *n* (%) | 11 (13) | 2 (11) | 9 (14) | 0.77 |
| Hyperlipidemia | 25 (30) | 2 (11 | 23 (35) | **0.04** |
| Pulmonary disease | 8 (10) | 0 (0) | 8 (12) | **0.04** |
| Obstructive sleep apnea | 11 (13) | 0 (0) | 11 (17) | **0.02** |
| Follow up in months; median (range) | 53 (0.7-238) | 9.6 (0.7-47) | 68.2 (11-238) | **<0.0001** |
| Deaths; *n* (%) | 5 (6) | 0 (0) | 5 (8) | 0.11 |

Abbreviations: WHO, World Health Organization; polycythemia vera, PV; Epo, erythropoietin; LDH, lactate dehydrogenase.

Body mass index was evaluable in n=2 idiopathic erythrocytosis patients (11%), and thus was excluded from analysis.
