# Supplementary material for: Phenotypical differences and thrombosis rates in secondary erythrocytosis versus polycythemia vera
Source: Blood Cancer J. 2021 Apr 15;11(4):75. doi: 10.1038/s41408-021-00463-x (PMC8050282; doi:10.1038/s41408-021-00463-x)
Supplement: Supplementary file 3 — Supplemental Table 3 [file 41408_2021_463_MOESM3_ESM.docx]

**Supplemental Table 3.** **Clinical and laboratory characteristics associated with thrombosis at any time in patients with secondary erythrocytosis versus World Health Organization-defined polycythemia vera**

| **Variables** | **All patients**  **(*n*=102)** | **Patients without thrombotic events**  **(*n*=69)** | **Patients with thrombotic events (n=33)** | **P value** |
| --- | --- | --- | --- | --- |
| Age at diagnosis, years; median (range) | 61 (19-89) | 58 (19-86) | 65 (36-89) | **0.001** |
| Age >50 years at diagnosis; *n* (%) | 74 (73) | 43 (62) | 31 (94) | **0.0003** |
| Males; *n* (%) | 57 (56) | 41 (59) | 16 (48) | 0.29 |
| Type of polycythemia: PV; *n* (%) | 66 (65) | 42 (61) | 24 (73) | 0.23 |
| Serum Epo levels at diagnosis, mIU/mL; median (range)  “N” evaluable=92 (90%) | 2.9 (<1-148) | 3.8 (<1-35) | 2.5 (<1-148) | 0.53 |
| Hemoglobin, g/L; median (range)  “N” evaluable=100 (98%) | 178.5 (151-223) | 178.5 (151-223) | 177.5 (151-214) | 0.69 |
| Hematocrit; median (range)  “N” evaluable=99 (97%) | 53.7 (44.6-70.2) | 53.5 (44.6-67) | 53.9 (44.7-70.2) | 0.4 |
| Hematocrit>55%; *n* (%)  “N” evaluable=99 (97%) | 39 (39) | 25 (37) | 14 (45) | 0.43 |
| Platelets, x 10^9^/L; median (range)  “N” evaluable=101 (99%) | 326 (120-995) | 302 (125-909) | 354 (120-995) | 0.49 |
| Platelets>450 x10^9^/L; *n* (%)  “N” evaluable=101 (99%) | 27 (27) | 17 (25) | 10 (31) | 0.49 |
| Leukocytes, x 10^9^/L; median (range)  “N” evaluable=101 (99%) | 8.9 (4.1-20.5) | 8.6 (4.1-15.1) | 9.3 (4.5-20.5) | 0.29 |
| Leukocytes>10x10^9^/L; *n* (%)  “N” evaluable=101 (99%) | 36 (36) | 25 (36) | 11 (34) | 0.86 |
| LDH at diagnosis, U/L; median (range)  “N” evaluable=72 (71%) | 216 (126-874) | 214 (126-874) | 230 (157-431) | 0.5 |
| Palpable splenomegaly at diagnosis; *n* (%)  “N” evaluable=97 (95%) | 16 (16) | 11 (17) | 5 (16) | 0.87 |
| Presence of *JAK2V617F* or exon 12 mutation *n* (%)  “N” evaluable=102 (100%) | 64 (63) | 41 (59) | 23 (70) | 0.31 |
| Active smoker; *n* (%) | 19 (19) | 13 (19) | 6 (18) | 0.94 |
| Hypertension; *n* (%) | 43 (42) | 19 (28) | 24 (73) | **<0.0001** |
| Diabetes; *n* (%) | 17 (17) | 7 (10) | 10 (30) | **0.01** |
| Obesity; *n* (%)  “N” evaluable=68 (67%) | 14 (20) | 5 (12) | 9 (36) | **0.01** |
| Hypercholesterolemia | 33 (32) | 13 (19) | 20 (61) | **<0.0001** |

Abbreviations: WHO, World Health Organization; polycythemia vera, PV; SE, secondary erythrocytosis; Epo, erythropoietin; LDH, lactate dehydrogenase; *JAK2,* Janus kinase 2.
